# Challenges and Opportunities in Radioligand Therapy

**DOI:** 10.2967/jnmt.125.270169

**Published:** 2025-12

**Authors:** Geoffrey M. Currie, Dale L. Bailey

**Affiliations:** 1 Charles Sturt University, Wagga Wagga, New South Wales, Australia;; 2 Baylor College of Medicine, Houston, Texas;; 3 Royal North Shore Hospital, Sydney, New South Wales, Australia; and; 4 University of Sydney, Sydney, New South Wales, Australia

**Keywords:** theranostics, radionuclide therapy, peptide receptor radionuclide therapy, PET, radioligand therapy

## Abstract

*Radioligand therapy* (RLT) is a recent term for long established nuclear medicine practices. Although radioiodine provides the historical foundations of theranostics, the prototype RLTs include ^68^Ga-DOTATATE and ^177^Lu-DOTATATE, which target somatostatin receptor subtype 2 in neuroendocrine tumors, and ^68^Ga-PSMA-617 and ^177^Lu-PSMA-617, which target prostate cancer. There are several challenges and opportunities in the realization of precision medicine through RLT. RLT, weaponized in cancer management through advances in instrumentation and radiochemistry, is transforming the nuclear oncology landscape. Equitable access to these advanced tools remains a global consideration.

Although there has been considerable attention given to the transformational nature of artificial intelligence (AI) in the field of nuclear medicine (1–3), it is theranostics that has recently produced seismic shifts in the nuclear medicine landscape (4,5). Advances in radiopharmacy, technology, and instrumentation ensure that the nuclear medicine industry is accustomed to seamless assimilation of change into clinical practice. Often, as is the case with theranostics, the underlying principle remains the same, but the tools and the metalanguage evolve. Theranostics is a portmanteau, combining parts of the words *diagnostic* and *therapeutic* to reflect principles dating back to radioiodine use in the 1940s (5–8). With change and advances come challenges and opportunities, with some of the key challenges and future or emerging opportunities discussed below.

## CHALLENGES IN THERANOSTICS

### Etymology, Linguistics, and Ontology

The term *theranostics* is used widely in the literature to represent a variety of pairs of diagnostic tools used to guide therapy. More conventionally, theranostic pairs are a single or similar entity for both diagnosis and treatment (9). This might include ultrasound or MRI for diagnosis and chemotherapy, radiotherapy or immunotherapy for therapy. In nuclear medicine and molecular imaging, theranostics is synonymous with PET paired with targeted radionuclide therapies using the same molecular probe (6–9). To avoid ambiguity and confusion, and to more accurately reflect what is being performed, the term *radioligand therapy* (RLT) might be more appropriate in nuclear medicine and molecular imaging (4). RLT, therefore, might be better defined as the pairing of diagnostic and therapeutic radioligands to optimize treatment outcomes and minimize toxicity to nontarget tissues (6–12).

The terms *theranostics* and *theragnostics* have been used interchangeably in the clinical setting and in the literature. Expert linguistic advice suggests that, although neither was consistent with Greek etymology, *theragnostics* was the least imprecise (13). If Greek etymology were the driver, the correct term might be *therapognostics* (4). The portmanteau, however, is a creative literary tool that does not follow either Greek or Latin language conventions. A portmanteau aims to combine multiple words in a way that shortens overall length but captures a sense of the meaning of each word (4). Either *theranostics* or *theragnostics* fits these requirements; however, *theranostics* is linguistically more acceptable and should be the preferred term, especially since *theragnostics* may be confused by the layperson to suggest “agnostic to therapy.”

With the emergence of RLT has come the growth and establishment of dedicated theranostic suites or laboratories. It is not uncommon for the theranostic suite to be the therapy suite or clinic where the radionuclide therapy is administered to patients, with the associated imaging being performed elsewhere in the nuclear medicine department. A theranostic suite by definition should be inclusive of the diagnostic and therapeutic facilities; otherwise, it is literally and figuratively a therapy suite.

RLT can allow *personalized medicine* while enhancing patient outcomes, which drives the concept of precision medicine. Nonetheless, the terms *personalized medicine* and *precision medicine* tend to be used interchangeably. Although personalized medicine focuses on individualizing a patient’s health care on the basis of their preferences and needs (individual nuance), precision medicine focuses on optimizing the outcomes for groups or subgroups of people. There are other dimensions and definitions of the terms, but these tend to be domain specific. For RLT, modifying imaging and therapy on the basis of individual social, cultural, economic, genetic, pathologic, and clinical factors may lead to less-than-optimal patient outcomes and challenge both evidence-based standardized approaches and precision medicine. Conversely, the use of nuances of biodistribution and organ function (e.g., kidneys) to customize patient doses to reduce toxicity and optimize target tissue dose is precision medicine.

With RLT comes an increase in data density and electronic information management. Information retrieval relies on standardization of metalanguage and attributes. RadLex is the lexicon of radiology, mapped and connected across the corpus of radiology ontology (14,15). Although originally developed for educational benefits, RadLex provides a standardized hierarchy of the language of radiology that benefits clinical and research practice (15). Much of RadLex translates to the nuclear medicine and molecular imaging ecosystem; however, there are niche terms and phrases used in theranostics and nuclear medicine that are not included in RadLex. The Society of Nuclear Medicine and Molecular Imaging AI Taskforce initiated the extension of RadLex to include key nuclear medicine terms and organize the lexicon in a hierarchal fashion under the banner of NucLex (16). NucLex is critical for facilitating data sharing, research, and development in RLT. Concurrently but independently, the Nuclear Medicine Global Initiative working group of the Society of Nuclear Medicine and Molecular Imaging has been working toward a standardized nomenclature for radionuclide therapy to create more uniform language globally (17).

### Chemistry of Theranostic Pairs

Since RLT pairs of diagnostic and therapeutic radionuclides share a common pharmaceutical or biomolecule (6–10), the physiologic behavior of each pair should be the same. The efficacy is optimized when the ligand selectivity and affinity for the target tissue are high (e.g., DOTATATE has high selectivity and affinity) and diagnostic efficacy and response to therapy are dependent on this receptor principle (4,10). That is, an overexpression of particular cell surface receptors or antigens can be targeted with a specific probe or ligand (18). Other than selectivity and affinity, ligands can have different potencies of action, inhibitory or stimulatory effects, and actions could be agonistic or antagonistic (e.g., prostate-specific membrane antigen [PSMA]–targeted ligands are antagonists; somatostatin-targeted ligands are agonists).

The radionuclide must be bound to the ligand without significantly altering its molecular behavior. Although direct labeling and prosthetic groups are used, RLT predominantly uses chelators to bind the radionuclide with a linker to connect the chelator to the ligand. This approach ensures the active portion of the ligand is not affected (7,10). Dodecane tetraacetic acid (DOTA) is a macrocyclic chelator most commonly associated with ^68^Ga, ^177^Lu, and ^225^Ac radionuclides and gadolinium contrast in MRI (4,5). Other common macrocyclic chelators including nonane triacetic acid (NOTA), tetradecane tetraacetic acid (TETA), and sarcophagine (4,5). Additionally, there are several common linear chelators, including diethylenetriaminepentaacetic acid (DTPA), hydroxybenzylethylenediamine diacetic acid (HBED-CC), and ethylene diamine tetramethylene acid (EDTA) (4,5).

The key issue is maintaining the in vivo integrity of both receptor affinity and ligand chelation. Although neither presents as an issue over the short timeline of diagnostic imaging, especially PET, where imaging for ^18^F and ^68^Ga typically occurs 1 h after administration, the longer physical and biologic half-lives of the therapeutic probe mean that dechelation, demetallation, and transmetallation of the radionuclide, or displacement of the probe from the target receptor/antigen, will produce nontarget radiation dosimetry (4,5,19). Furthermore, this biologic redistribution and associated variability in dosimetry would be difficult to predict on the basis of diagnostic imaging. For therapeutic radionuclides with γ-emissions suitable for imaging (e.g., ^177^Lu), serial imaging for 5 d or more after dose administration may allow for dosimetry estimates, which could be used to adjust the dose for subsequent cycles (4). This luxury is not afforded to pure particle emissions (e.g., ^90^Y, ^225^Ac).

**FIGURE 1.**
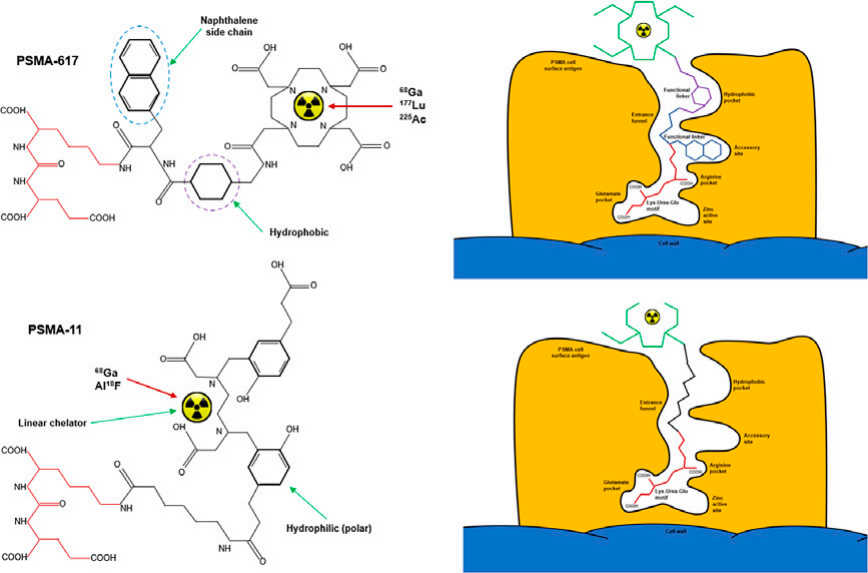
Schematic representation of PSMA-targeted peptides, showing common active pharmacophore (red). PSMA-617 (top) is macrocyclic (DOTA) chelator. PSMA-11 (bottom) is linear chelator (HBED-CC). Naphthalene side chain and hydrophobic ring of PSMA-617 increases binding affinity absent in PSMA-11. Image reproduced with permission (5).

For authentic RLT pairs, diagnostic and therapeutic probes need to reflect the identical molecular pathway. For example, ^68^Ga-DOTATATE and ^177^Lu-DOTATATE both have DOTA as the chelator and TATE (octreotate) as the peptide. Conversely, ^64^Cu-SARTATE has the same peptide but adopts a different chelator, which produces subtle variations in biodistribution (4,5). Across the range of PSMA-targeted probes, the pharmacophore, or active portion of the peptide, remains the same, whereas important secondary features of the peptide and the chelators vary substantially (4,5). PSMA-617 has 2 additional accessory pockets for high-affinity antigen binding combined with the macrocyclic DOTA chelator. Conversely, PSMA-11 has lower-affinity antigen binding in the absence of accessory binding sites and is more susceptible to demetallation because it adopts the HBED-CC linear chelator (Fig. 1). Pairing ^68^Ga or ^18^F-PSMA-11 with ^177^Lu-PSMA-617 is not a true theranostic pair. It is also worth noting that ^18^F-PSMA-11 adopts pseudoradiometal chelation by using secondary binding to aluminum, further altering molecular behavior (4,5). There are 3 general classifications of RLT pairs (20): the same radionuclide for imaging and therapy (e.g., ^131^I), different radionuclides of the same element for imaging and therapy (e.g., ^123^I and ^131^I), and radionuclides of different elements for imaging and therapy (e.g., ^68^Ga and ^177^Lu).

Gallium is a trivalent earth metal in group 13 of the periodic table, lutetium is a group 3 transition metal in the lanthanide series, and actinium is a group 3 transition metal in the actinide series (5). Among gallium, lutetium, and actinium, there are variations in complexity of coordination chemistries, different coordination geometries, and different formation constants, which result in variations in their binding, biodistribution, and stability (21,22). For example, when adopting the same chelator and ligand (e.g., HBED-CC PSMA-11), ^68^Ga produces simpler chemistry and a 3^+^ charge compared with the more complex chemistry and 2^+^ charge of Al^18^F, which alters pharmacokinetics and biodistribution (23). Although different radionuclides of the same element optimize coordination chemistry between the diagnostic and therapeutic radioligand pair, it is possible that the small differences in atomic mass can produce small variations because of the mass-dependent isotope effect, which may alter chemical or physical process (e.g., diffusion) (5). Of specific interest among the mass-dependent isotope effects are the *kinetic isotope effects* that result in changes to the association rates and dissociations rates of chemical reactions. For pairs like ^123^I/^131^I and ^67^Cu/^64^Cu, small variations in biodistribution may result, particularly for more complex radioligands (5).

The variable physical properties (emission type, energy, and half-life) of diagnostic and therapeutic pairs, even with identical peptide and chelator structures, may have different chemical properties (24). It may be simple to consider ^68^Ga and ^177^Lu (and even ^225^Ac) as sharing the chemical properties of group 3 radiometals but this is not accurate. Although sharing some properties that make radiochemistry similar, the differences may have an impact on the half-life of therapy. True theranostic pairs will also share the same element (e.g., ^123^I/^131^I) and are driving research in radionuclide development (e.g., ^86^Y/^90^Y and ^44^Sc/^47^Sc theranostic pairs).

### Access and Equity

The future of RLT confronts the challenge associated with radionuclide production capacity and radionuclide costs (19,25). Global commercial production of ^177^Lu is currently limited, and although production is expected to triple globally for ^177^Lu by 2033 (25), this anticipated production rate falls far short of the predicted growth in ^177^Lu demand (19,26,27). At the same time, demand for ^68^Ga continues to increase beyond the supply limits of ^68^Ge for generators (19). Cyclotron production of ^68^Ga may be increasingly required to meet global demand, which may disadvantage clinical sites without onsite cyclotron production (i.e., its 68-min half-life presents transport barriers) if ^68^Ge/^68^Ga generators are limited in supply (19,28,29). It should be noted, however, that there are several limitations to cyclotron production related to the short half-life and requisite radiochemical and radionuclidic purification and testing. The more abundant ^18^F may have an increasing role in creating flexible patient volumes and scheduling.

Globally, there are significant disparities in access to RLT attributable to socioeconomic, geographic, and medical insurance/reimbursement factors (19,25,30). The typically short half-life of PET radionuclides can increase barriers in developing economies and geographically isolated communities, where local production is cost-prohibitive. The cost of production and transport for therapeutic radionuclides can also be cost-prohibitive (19). Improving access to RLT may require cost efficiencies, new radionuclides more suitable for longer transport times, SPECT-based imaging using ^99m^Tc, and novel generator availability for theranostic pairs (e.g., ^99^Mo/^99m^Tc imaging paired with ^188^W/^188^Re for therapy).

## EMERGING AND FUTURE OPPORTUNITIES FOR RLT

### Radiation Dosimetry

**FIGURE 2.**
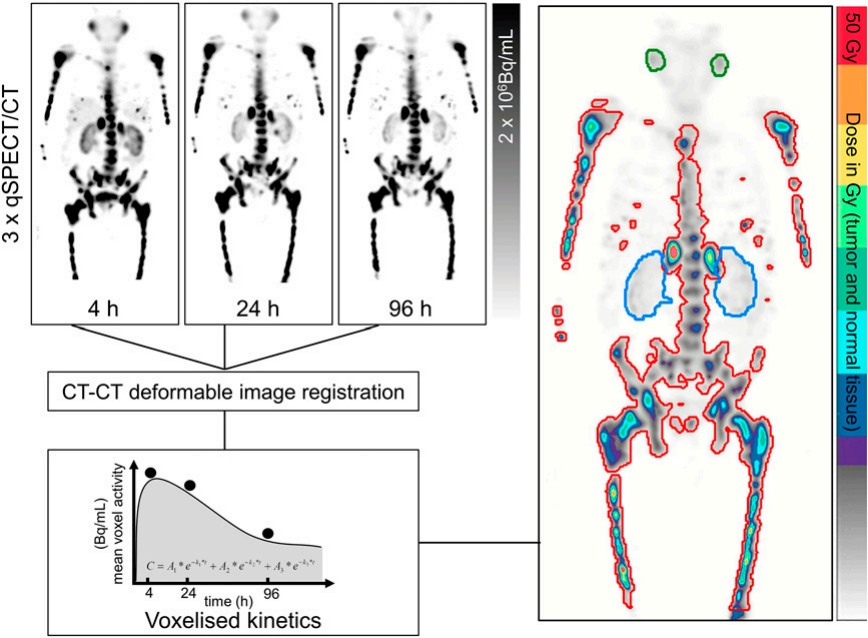
Schematic of voxel-based dosimetry workflow using regions of interest for whole-body tumor volume (red), kidneys (blue), and salivary glands (green) for kinetic modeling. Reprinted with permission (34).

Although PET imaging is the best predictor of therapeutic outcomes of RLT (31), imaging could also provide important insights into dosimetry (4,32,33). Therapeutic radionuclides with a γ-emission suitable for imaging (e.g., ^177^Lu) allow for serial posttherapy images that can be used for radiation dosimetry calculations (Fig. 2) in target and nontarget tissues, independently of whether the diagnostic/therapeutic pair is a true theranostic (10,18,34). In theory, this could allow adjustments to the dose given in subsequent therapy cycles to optimize tumor dose burden and minimize nontarget tissue dose burden. In practice, more standardized approaches (fixed dose) to the therapeutic regimen, which tend to be based on radiation dose rather than absorbed dose, are generally recommended (31,35,36). Personalizing therapy doses based on dosimetry would be more consistent with the precision medicine mantra. Not all therapeutic radionuclides allow adequate γ-imaging (e.g., ^90^Y and ^225^Ac). There have been efforts to produce images from therapy doses using bremsstrahlung imaging, PET imaging after pair production, and Cerenkov luminescence (37), but these images are of poor quality and are not suitable for accurate radiation dosimetry calculations.

Proactive radiation dosimetry using diagnostic images could predict the likely distribution of the therapeutic tracer (12), but the accuracy of this approach is constrained by several barriers. First, there may be variations in molecular behavior between the theranostic pairs that are not true theranostic pairs, as discussed above. Second, the typically short half-life of the diagnostic radionuclide and the use of imaging 60 min after administration does not adequately map the biodistribution of the therapeutic radionuclide over several days. A potential solution is to perform serial images using a test dose (low dose) of the therapeutic radionuclide (36). Alternatively, a longer-lived diagnostic radionuclide could be used (e.g., ^64^Cu, ^66^Ga, and ^89^Zr). Dual-time-point imaging of short-lived diagnostic radionuclides may provide richer insights than that of a single time point, and pharmacokinetic data using the higher sensitivity of long–axial-field-of-view PET could also improve predictive dosimetry. Others have used Monte Carlo simulations from ^68^Ga PET imaging for predictive dosimetry of ^177^Lu therapy (38). The emergence of deep-learning algorithms is likely to reshape this landscape.

Dual-time-point diagnostic imaging is not a new concept and has been used to enhance semantic interpretation (39–41), such as delineating a small lesion from background activity. The opportunity to provide richer insights for predictive dosimetry is important. The longer half-life of ^18^F over ^68^Ga provides an improved opportunity for dual-time-point imaging. The increasing sensitivity of PET scanners, particularly long-bore PET scanners, afford the opportunity for dual-time-point imaging with short-lived radionuclides at significantly later times (e.g., ^68^Ga imaged at 4 h and ^18^F at 10 h) to improve the predictive accuracy of dosimetry calculations and produce better target-to-background ratios.

Long-lived imaging radionuclides, such as ^64^Cu (12.7 h plus a β-minus emission) and ^89^Zr (78.4 h, which is longer than ideal), would also allow delayed imaging (42). Unfortunately, variations in coordination chemistries and radionuclide chelation means theranostic pairs may have different molecular behaviors over time and undermine the accuracy of radiation dosimetry calculations. For example, ^68^Ga chelates with DOTA as an N_4_O_2_ donor, ^177^Lu to DOTA as a N_4_O_3_ donor, and ^225^Ac to DOTA as a N_4_O_4_ donor (21,43–46), whereas ^64^Cu chelates to a sarcophagine as a N_6_ donor and ^89^Zr as an O_8_ donor to chelators such as deferoxamine (linear) and fusarinine (macrocyclic) (22,47). ^66^Ga might be a suitable PET imaging option, with a 9.5-h half-life, but relies on cyclotron production rather than the convenience of a generator (42).

**FIGURE 3.**
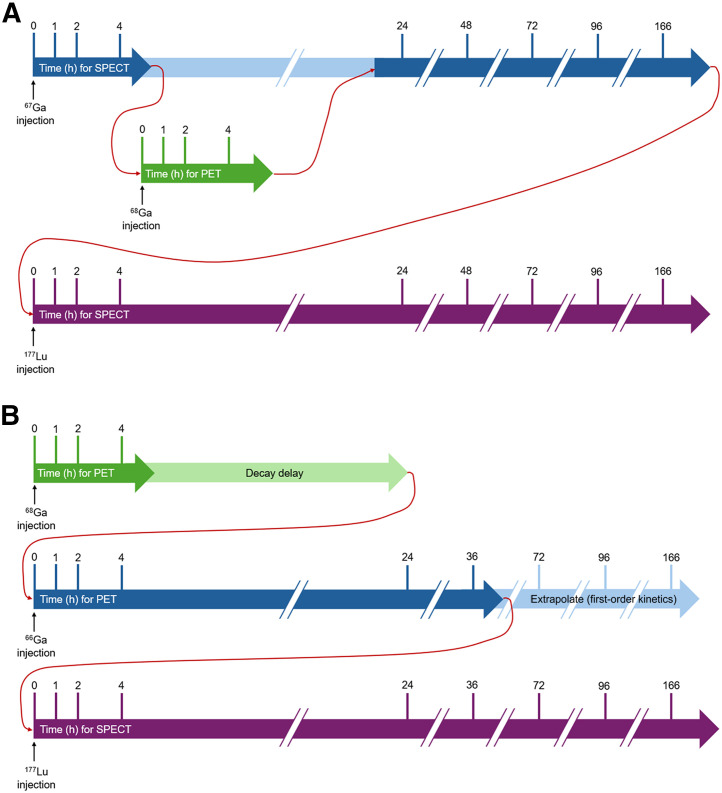
Potential validation protocol for ^67^Ga/^177^Lu theranostics (A) and ^66^Ga/^177^Lu theranostics (B).

A novel approach could be developed that incorporates several of the principles outlined above. Consider first that predictive dosimetry from the PET scan is generally limited by the short half-life in predicting the SPECT-based distribution of the longer-lived therapeutic radionuclide (48). There is little semblance in image quality between ^68^Ga-based PET and ^177^Lu-based SPECT, for example. ^67^Ga, despite facing production and cost barriers at the time of writing, will chelate identically to ^68^Ga (subject to the kinetic isotope effect of a single neutron difference), which could not only allow serial delayed imaging (78-h half-life) but also use γ-imaging with SPECT to more closely resemble the serial ^177^Lu imaging. Whole-body imaging of ^67^Ga-labeled ligand administered at time 0 could be performed at 1, 2, and 4 h after injection. After the 4-h imaging, the same ligand labeled to ^68^Ga could be administered and imaged with long–axial-field-of-view PET at 1, 2, and 4 h after injection. The PET images could be used to validate the SPECT images. Subsequently, the ^68^Ga decay would allow interference-free ^67^Ga imaging at 24, 48, 72, 96, and 166 h after ^67^Ga injection, which could be used to predict radiation dosimetry to target and nontarget tissues. After ^177^Lu-labeled radioligand administration, whole-body ^177^Lu images could be performed at 1, 2, 4, 24, 48, 72, 96, and 166 h after ^177^Lu administration as the ground truth for validation of the ^67^Ga dosimetry (Fig. 3). One might expect that, once validated, fewer serial imaging time points will be required to build an accurate radiation dosimetry profile that could be used to optimize and individualize therapy doses before administration—proactive rather than reactive radiation dosimetry consistent with precision medicine. The ^67^Ga approach has been described with use in DOTA-miltuximab prostate cancer theranostics (48). It is possible that variability in pharmacokinetics for critical organs and tissues predominates in the first 24–36 h after ^177^Lu administration, after which more predictable first-order kinetics could be applied. In this case, ^66^Ga PET might provide an alternative approach to ^67^Ga SPECT in a truncated version of the same protocol outlined above. Although further evaluation is required, this approach could overcome the barriers confronting current modeling approaches and machine-learning/deep-learning algorithms.

### AI

The emergence of AI and deep-learning algorithms could be transformational in the RLT space. AI is likely to enhance precision theranostics with improvements in workflow, data management, predictive radiation dosimetry, and data curation. Although AI applications in radiology, radiation oncology, and nuclear oncology have been widely reported, there are exciting opportunities in RLT to be explored. These applications and opportunities are discussed elsewhere in this special edition (49).

### Novel Radionuclides

A wide range of new radionuclides is being explored and developed for application in RLT (5). Although cost and availability are important considerations, optimizing fitness for purpose is the principal driver. As previously discussed, increased demand has led to challenges related to the cost and availability of ^68^Ga and ^177^Lu. Limitations for ^68^Ge/^68^Ga generators, for example, have resulted in the emergence of cyclotron production of ^68^Ga and substitution with the more widely available and less expensive ^18^F. The physical characteristics of diagnostic and therapeutic radionuclides are critical (i.e., emission type, energy, abundance, and half-life). For PET imaging, the half-life should match the imaging window and have a suitable abundance and energy of the positron emission. For the therapeutic radionuclide, a pure particle emitter could be viewed as an advantage by allowing a large patient dose with little external radiation hazard. Conversely, a particle emission with associated γ-emissions of an energy and abundance suitable for imaging (PET or SPECT) would allow postdose serial images for biodistribution and dosimetry. Additionally, a therapeutic radionuclide should have a physical half-life matched to retention in diseased tissue, and the type of particle (α or β) and its energy should match the tissue penetration that optimizes target tissue dose burden and minimizes potential injury to nontarget tissues (e.g., α-therapies for micrometastatic disease).

The concept of true theranostic pairs was discussed previously and emphasized the value of diagnostic and therapeutic radionuclide pairs that share the same chemistry to avoid differences in biologic behavior attributable to chelation, coordination chemistry, or stability. The prototype theranostic is either ^131^I as both the diagnostic and therapeutic pair or ^123^I/^124^I for imaging and ^131^I for therapy because they are the same element. Emerging theranostic pairs that share the same element include ^64^Cu/^67^Cu, ^86^Y/^90^Y, and ^44^Sc/^47^Sc. Other than the aforementioned ^131^I and ^177^Lu, ^149^Tb and ^153^Sm are radionuclides that can be used in imaging and therapy but generally are suboptimal for diagnostic imaging or therapy (or both). In the absence of pairs of the same element, significant effort has been made toward investigating theranostic pairs of elements from the same group of the periodic table to more closely align their chemical behavior. Perhaps the most widely used and investigated are the group 3 (including lanthanides and actinides) therapeutic radionuclides, such as yttrium, samarium, holmium, erbium, lutetium, and actinium, with the group 13 earth metals (e.g., gallium, indium) because they behave as trivalent metals.

Considerable interest has emerged recently with α-emitting lanthanides (e.g., ^149^Tb) and actinides (e.g., ^227^Th, ^225^Ac), and radionuclides that are a product of their complex decay schemes (e.g., ^213^Bi, ^211^At, ^212^Pb) (22,50). The very high linear energy transfer of α-particles increases double-strand DNA damage while reducing cross-fire damage (46,50,51). Unfortunately, this can also increase damage to nontarget tissues (e.g., kidneys during elimination). This is compounded by short half-lives with decay to daughter products with their own β-or α-emissions and whose properties can be sufficiently different to allow dechelation/transmetallation/demetallation, associated with altered biodistribution and subsequent nontarget tissue dose (50,51). For example, ^212^Pb has very different coordination geometry from the daughter ^212^Bi, which results in 30% dechelation on decay (50). The current cost of α-therapies combined with toxicity risks is prohibitive of widespread clinical use (50,51). An interesting radionuclide for PET is ^94m^Tc (52-min half-life), which would effectively radiolabel to any ^99m^Tc-based ligands used in SPECT.

For those without access to PET-based theranostics due to the previously discussed socioeconomic or geographic factors, there is a pressing need for SPECT-based theranostics. SPECT imaging is typically undertaken with ^99m^Tc because the ^99^Mo/^99m^Tc generator provides cost-effective services remotely from production sites. There can be several barriers to the use of therapeutic probes. The first relates to the need for onsite *synthesis*, which is more complex than the cartridge-and-kit-based radiopharmacy typical of ^99m^Tc radiolabeling. Sites without a radiopharmacist or radiochemist may not be able to *manufacture* the finished probe, even if the unbound therapeutic radionuclide could be delivered. Therein lies the second issue. Therapy dose delivery (e.g., ^177^Lu) has increased costs related to its transport to remote areas, the dose suffers decay losses during longer transit times, and this treatment has a higher risk burden if a patient misses or cancels their appointment. Solutions should include delivery of a ready-for-use end product (e.g., ^177^Lu-chelated PSMA-617), radionuclides with kit-based chemistry that mirrors ^99m^Tc (e.g., ^186^Re), and radionuclides available via generator (e.g., ^188^Re).

### Novel Theranostic Ligands

Over the past few years, the principle of theranostics has been synonymous with ^68^Ga- DOTATATE,^177^Lu-DOTATATE, ^68^Ga-PSMA-617, and ^177^Lu-PSMA-617. The future of RLT will see the emergence of a variety of novel ligands. Although there are several exciting new RLT pairs being developed or evaluated, there have also been radiochemical improvements to our prototype theranostics that enhance pharmacokinetics and safety or incorporate a novel radionuclide. ^225^Ac-DOTATATE is a widely reported somatostatin subtype receptor 2 agonist (52–54), but ^225^Ac has also been coupled to somatostatin subtype receptor 2 antagonists such as DOTA-LM3, which has been shown to be effective in ^18^F-FDG avid disease that lacks ^68^Ga-DOTATATE avidity (55), and has been imaged with high avidity with antagonists such as ^68^Ga-NODAGA-LM3 or ^68^Ga-DOTA-LM3 (55). Somatostatin antagonists have lower levels of internalization, greater numbers of receptor-binding sites, and high affinity for receptors, compared with agonists, and have been coupled with ^68^Ga, ^64^Cu, ^177^Lu, and ^225^Ac (56). There are also novel applications of DOTATATE. In Australia, for example, the recent Medicare coverage (government funding) approval for ^177^Lu-DOTATATE therapy included a provision for use in neuroendocrine tumors or other tumors with higher somatostatin expression (e.g., renal cell carcinoma, breast cancer, meningioma). ^225^Ac-DOTATATE has been used in conjunction with PET imaging for the treatment of metastatic breast cancer (57).

**FIGURE 4.**
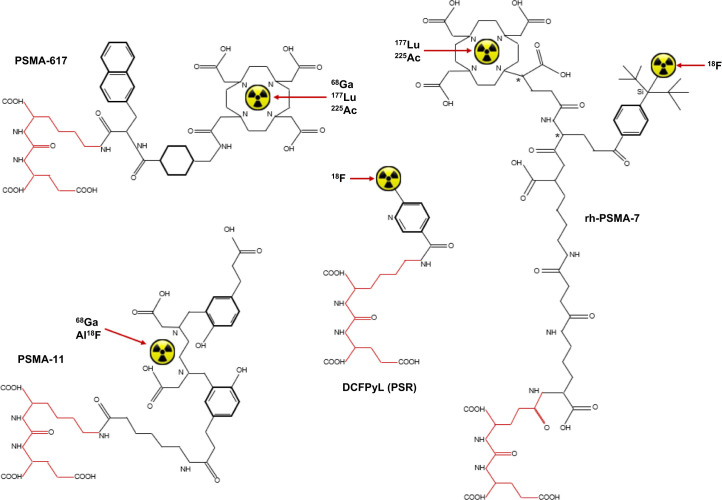
Schematic representation of chemical structures of key PSMA-targeted ligands, including DOTA-chelated PSMA-617, HBED-CC-chelated PSMA-11, direct ^18^F labeling of DCFPyL, and Si^18^F acceptor moiety of rh-PSMA-7, with potential DOTA chelation of therapeutic radionuclides. * indicates diastereomeric mixture.

There have also been developments with PSMA-targeted ligands. ^225^Ac-DOTA-PSMA has been paired with ^68^Ga-DOTA-PSMA imaging (58), and Sar-bis-PSMA with ^64^Cu/^67^Cu pairings have also been reported (22). Several PSMA-targeted monoclonal antibodies have been developed as theranostic pairs using ^68^Ga- and ^177^Lu-rosopatamab (TLX591) or ^177^Lu-TLX591 therapy paired with ^68^Ga-PSMA-11 or ^18^F-PSMA-11 imaging (59). Mitigating the Al^18^F linear chelator limitations of PSMA-11, the development of ^18^F-radiohybrid PSMA-7.3 (^18^F-rhPSMA-7), or flotufolastat, uses the same active pharmacophore as other PSMA-targeted ligands but adopts a silicon fluoride acceptor moiety in a diastereomeric mixture (nonmirrored, nonidentical stereoisomers) that can be paired with ^177^Lu-rhPSMA-7 (60–62) as a more authentic theranostic pair than ^18^F-PSMA-11 and ^177^Lu-PSMA-617 (Fig. 4). An interesting potential application of this approach, including Si^18^F-TATE for somatostatin targeting, is that the chemical structure includes a DOTA chelator that could be used for simultaneous therapy (63). The short half-life of ^18^F would limit the usefulness of serial posttherapy dosimetry PET imaging for therapeutic radionuclides without γ-emissions (e.g., ^225^Ac), although the combination with high-sensitivity long–axial-field-of-view PET scanners may allow imaging 24 h after administration. PSMA-617 has also been chelated to the β-, Auger, and Coster-Kronig electron-emitting ^161^Tb for metastatic prostate cancer therapy and showed superiority over ^177^Lu-PSMA-617 in animal and human trials (64,65) while the VIOLET trial of ^161^Tb-PSMA-I&T is recruiting patients at the time of writing (66).

Beyond these prototype theranostics, there is a range of new and innovative theranostics, with the literature expanding rapidly. At the time of writing, several were in development or under clinical evaluation. Girentuximab is a carbonic anhydrase 9–targeted monoclonal antibody with ^89^Zr PET imaging paired with ^177^Lu or ^225^Ac therapy in renal cell carcinoma (67). Theranostic pairing of ^64^Cu and ^67^Cu Sar-bombesin has been used to target gastrin-releasing polypeptide in a variety of cancers (22,51). Although bombesin can be chelated with a variety of SPECT, PET, and therapeutic radionuclides, the in vivo stability of the copper-chelated sarcophagine allows delayed imaging, improves predictive dosimetry, and reduces demetallation/transmetallation.

Significant interest has emerged for fibroblast activation protein inhibitor (FAPI) theranostics because of the high tumor-to-background uptake ratio (68). Both ^18^F-FAPI and ^68^Ga-FAPI have been paired with ^90^Y-FAPI and ^177^Lu-FAPI for the treatment of a variety of pathologies (10,51). There are, however, variations in biodistribution and associated dosimetry for different versions of FAPI (e.g., FAPI-4, FAPI-46, FAPI-2286) (10,51). These factors, combined with unfavorable biology of tumors overexpressing fibroblast activation protein (e.g., rapid tumor clearance), have slowed the transition from exciting imaging agent to realized theranostic potential (68,69). The less-than-ideal tumor retention of the therapy probe was addressed recently using albumin-binding moieties to increase tumor retention (69).

Both ^68^Ga/^90^Y and ^68^Ga/^177^Lu conjugation with pentixather have been used as theranostic pairs targeting chemokine receptor 4 associated with proliferation and angiogenesis (7,51). ^123^I metaiodobenzylguanidine can be paired with the α-emitting ^211^At meta-astatobenzylguanidine to target neural crest tumors (70). Overexpression of CD38 in lymphoma, multiple myeloma, and leukemia can be targeted with theranostic pairs of the monoclonal antibodies daratumumab or isatuximab with a variety of imaging radionuclides (^68^Ga, ^64^Cu, ^99m^Tc, ^89^Zr) and therapeutic radionuclides (^177^Lu, ^225^Ac, ^90^Y, ^212^Pb, ^211^At) (71). Breast cancer remains an important theranostic target, but despite a long history of molecular targeted therapies (e.g., estrogen receptor downregulators and human epidermal growth factor receptor 2 antagonists), progress on theranostic pairs has been slow (72). There are several promising theranostic approaches in breast cancer including, but not limited to, ^18^F/^68^Ga imaging combined with ^177^Lu/^225^Ac therapy conjugated with DOTATATE and FAPI (57,73,74). Overexpression of human epidermal growth factor receptor 2 allows theranostic targeting with the monoclonal antibody trastuzumab (51,74) in cancers of the breast, ovary, endometrium, bladder, lung, and gastrointestinal tract (51) using DOTA chelation of ^64^Cu, ^89^Zr, ^68^Ga and ^177^Lu (75). The success of estrogen receptor imaging with ^18^F-fluoroestradiol has not translated to theranostics.

### SPECT Theranostics

SPECT already plays an important part in the theranostics landscape with serial ^177^Lu imaging for radiation dosimetry; however, improved SPECT technology also makes evaluation of treatment response possible (76). Although PET offers superior spatial resolution, contrast, and quantitative accuracy compared with SPECT, the impact on outcomes, such as diagnosis, staging, and predicting response to radionuclide therapy, is not as clearly defined. For example, comparing ^68^Ga-PSMA PET with ^99m^Tc-PSMA SPECT, sensitivity variation may arise from lesion-by-lesion comparison that favors the spatial resolution of PET rather than impact on patient management. In a comparison of ^99m^Tc-HYNIC-iPSMA SPECT and ^68^Ga-PSMA-11 PET, the clinical utility and quantitative analysis of both were comparable (77). Head-to-head comparison of ^99m^Tc-HYNIC-iPSMA SPECT and ^68^Ga-PSMA PET indicated that SPECT had 100% sensitivity for lesions larger than 10 mm but only 28% for lesions smaller than 10 mm (78). The longer physical half-life (6 h) of ^99m^Tc could be used for delayed imaging to improve contrast and detection of smaller lesions (79). ^67^Ga has a 78.3-h half-life, with γ-imaging using 93, 184, and 300 keV emissions of 39%, 21%, and 17% abundance, respectively (48). As previously outlined, there is an opportunity to use ^67^Ga as an alternative to the short-lived ^68^Ga in radioligands to allow predictive dosimetry and more individualized therapeutic doses (48).

Given the previously discussed production capacity, cost, distribution barriers, and availability of ^68^Ga and ^177^Lu, and the socioeconomic and geographic disparities in access and availability to PET-based theranostics, the development of SPECT-based theranostics is essential. The radiochemical versatility of ^99m^Tc, suitability of ^99m^Tc for SPECT imaging (photon energy and half-life), and the supply efficiencies of the ^99^Mo/^99m^Tc generator, combined with advances in SPECT technology, provide an excellent imaging base for theranostics. Matching this versatility and convenience with generator-produced ^188^Re (^188^W/^188^Re) for radionuclide therapy would also exploit the near-identical radiochemistry shared between technetium and rhenium (19). Indeed, the challenge for SPECT-based theranostics is to develop authentic theranostic pairs. Other than the historical prototype theranostic pairing of radioiodine nuclides, ^99m^Tc and ^188^Re are radiochemically closer than ^68^Ga and ^177^Lu. Despite this, ^99m^Tc/^188^Re are seldom referenced in the development of new theranostic pairs.

**FIGURE 5.**
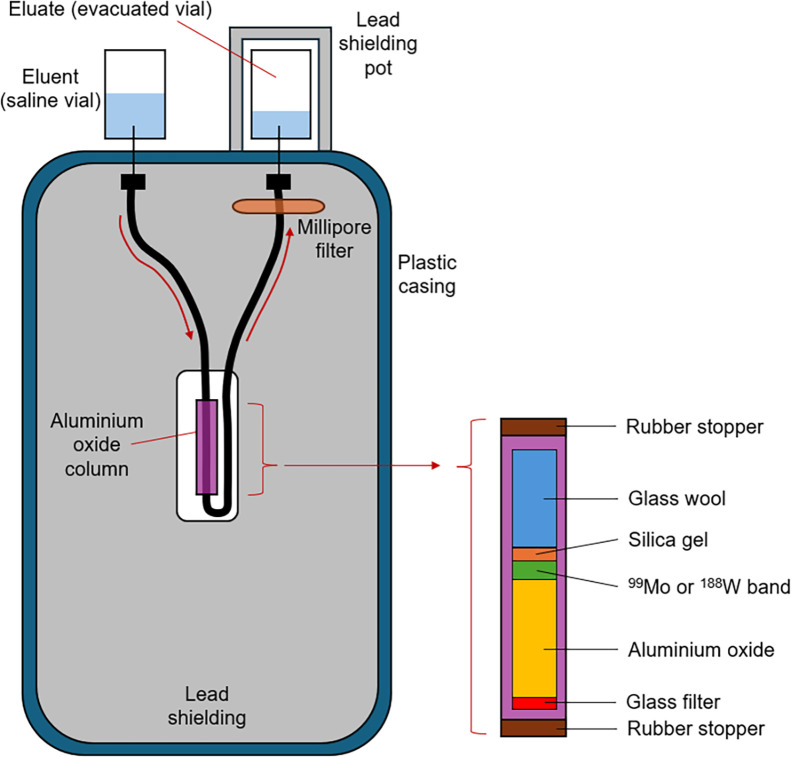
Schematic representation of shared design principles of ^188^W/^188^Re and ^99^Mo/^99m^Tc generators. Adapted and reproduced with permission (19).

In RLT, technetium and rhenium produce high chelation stability and versatile coordination chemistry. The ^99^Mo/^99m^Tc generator has a shelf-life of 1 wk (with potential use of 2 wk) and is eluted once or twice daily to maximize the elution profile. The 66-h half-life of ^99^Mo puts the ^99^Mo/^99m^Tc generator in transient equilibrium (80,81). The ^188^W/^188^Re generator has a shelf-life of about 9–10 mo and can be eluted daily. The 69.4-d half-life of ^188^W and 17 h for ^188^Re puts the ^188^W/^188^Re generator in secular equilibrium (82). The ^99^Mo/^99m^Tc and ^188^W/^188^Re generators operate on the same design principle (Fig. 5). It should be noted that ^186^Re is an excellent alternative to ^188^Re but is not available in a generator. ^188^Re has a 2.12 MeV β-emission and a 155 keV γ-emission (15.5%), whereas ^186^Re has a 3.7-d half-life, 1.07 MeV β-emission, and 137 keV γ-emission (9.5%) (19).

Historically, ^99m^Tc-chelated peptides lacked the specific targeting expected in theranostics (e.g., ^99m^Tc-apcitide, ^99m^Tc-HYNIC-Annexin V, ^99m^Tc-depreotide). More recent advances have seen the emergence of several ^99m^Tc/^188^Re based theranostics (some limited to preclinical evaluation), including ^99m^Tc and ^186/188^Re HYNIC-bombesin to target integrin α_v_β_3_ and gastrin releasing peptide receptor (GRPR) (83), ^99m^Tc and ^188^Re microspheres for selective intraarterial radionuclide therapy associated with hepatocellular carcinoma (84), ^99m^Tc and ^188^Re DMSA(V) for medullary carcinoma of the thyroid and breast cancer metastases in the liver, brain, and bone (85,86), and ^99m^Tc and ^188^Re anti-CD66 monoclonal antibody in leukemia and lymphoma (87). Radioimmunotherapy potential has also been shown for ^99m^Tc and ^188^Re anti-CD20 (rituximab) in lymphoma, ^99m^Tc and ^188^Re trastuzumab for breast cancer, ^99m^Tc and ^188^Re bevacizumab in non–small cell lung cancer, ^99m^Tc and ^188^Re cetuximab in lung cancer, ^99m^Tc and ^188^Re anti-EGFR antibody h-R3 (nimotuzumab) in glioma, and ^99m^Tc and ^188^Re anti-CEA MN-14 antibody in gastrointestinal cancers (86). Research has shown the potential pairing of ^99m^Tc and ^188^Re for α_v_β_3_ integrin, NK1 receptors, and VEGFR (86). ^99m^Tc FAPI-34 has been produced with high affinity and significant tumor uptake and may present a future opportunity for ^188^Re therapy (88).

**FIGURE 6.**
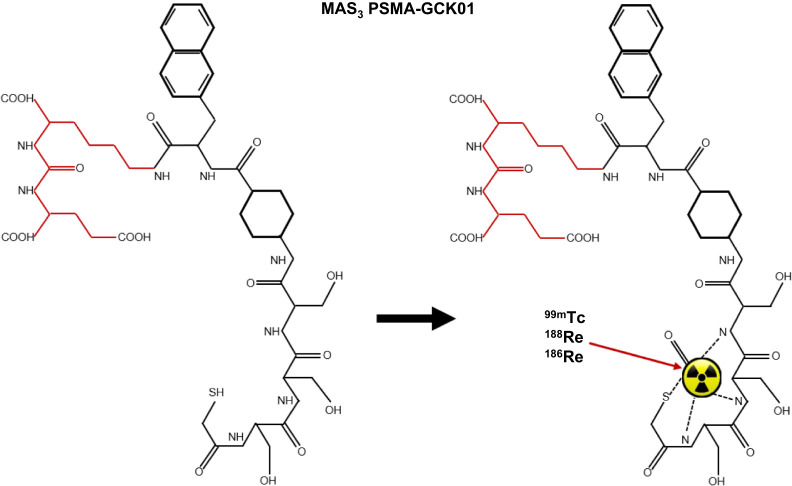
Mercaptoacetyl triserine (MAS_3_)–chelator used for technetium- or rhenium-chelated PSMA-GCK01. Red domains are active pharmacophore, which are identical across range of PSMA-targeted biomolecules (Lys-Urea-Glu motif).

While recent ^99m^Tc-EDDA-HYNIC-iPSMA has provided excellent imaging outcomes (30), nonspecific accumulation makes ^188^Re therapy less ideal. This limitation has been overcome using novel chelators that are also more suited to ^188^Re or ^186^Re chelation. Among them, the mercaptoacetyl triserine (MAS3)–chelated ^99m^Tc PSMA-I&S shows promise (89). Indeed, the ^99m^Tc/^188^Re MAS3-chelated PSMA-GCK01 theranostic pair (Fig. 6) provided targeted distribution and serial imaging for dosimetry and compared well with ^177^Lu PSMA-617 (90).

The original theranostic was the application of ^131^I sodium iodide for both imaging and therapy in thyroid disease, although production methods at the time meant a cocktail of both ^131^I and ^130^I (7). With an 8-d half-life and 364 keV γ-emission, ^131^I is less than ideal for imaging with a conventional γ-camera. A better theranostic pair uses ^123^I as the imaging radionuclide with its 13-h half-life and 159 keV γ-emission. ^124^I PET has been used for radioiodine theranostics to provide imaging and predictive dosimetry for ^131^I therapy (91). For theranostics, radioiodine has several applications (51,92):

- ^123^I sodium iodide/^131^I sodium iodide for thyroid cancer and metastatic thyroid carcinoma,
- ^123^I sodium iodide/^131^I sodium iodide for hyperthyroidism,
- ^123^I and ^131^I metaiodobenzylguanidine for a variety of neuroendocrine pathologies, including neuroblastoma, pheochromocytoma, paragangliomas, medullary thyroid carcinoma, and neuroendocrine tumor,
- ^131^I tositumomab to target the cell surface antigen CD20 that is overexpressed in non-Hodgkin lymphoma,
- ^123^I and ^131^I MIP-1072 for prostate cancer,
- ^123^I and ^131^I BA52 for metastatic melanoma, and
- ^123^I metaiodobenzylguanidine paired with ^211^At meta-astatobenzylguanidine to target neural crest tumors.

Several tumors have shown upregulation of the natrium iodine symporter, which provides some potential for sodium iodide in both ^124^I/^131^I and ^123^I/^131^I theranostics (90,92). Among them, breast cancer may be a beneficial target (91,93).

## CONCLUSION

Advances in both instrumentation and radiochemistry have reengineered PET-based RLT. With each advance there is a move closer to precision medicine but also an introduction of a new set of challenges to overcome. At the same time, it is important to not overlook capabilities of long-standing radionuclides and SPECT. To date, RLT has been efficacious in niche applications, as standardized dosing is independent of the authenticity of the theranostic pair (i.e., true theranostic). Converging on genuine precision medicine will demand developments in predictive theranostics and novel theranostic pairs or better use of existing capabilities (e.g., ^67^Ga). ^99m^Tc/^188^Re theranostics could address inequity in access to cutting-edge theranostics.

## DISCLOSURE

No potential conflict of interest relevant to this article was reported.
